# High Incidence of Chikungunya Virus and Frequency of Viremic Blood Donations during Epidemic, Puerto Rico, USA, 2014

**DOI:** 10.3201/eid2207.160116

**Published:** 2016-07

**Authors:** Graham Simmons, Vanessa Brès, Kai Lu, Nathan M. Liss, Donald J. Brambilla, Kyle R. Ryff, Roberta Bruhn, Edwin Velez, Derrek Ocampo, Jeffrey M. Linnen, Gerardo Latoni, Lyle R. Petersen, Phillip C. Williamson, Michael P. Busch

**Affiliations:** Blood Systems Research Institute, San Francisco, California, USA (G. Simmons, K. Lu, N.M. Liss, R. Bruhn, M.P. Busch);; University of California, San Francisco (G. Simmons, M.P. Busch);; Hologic, Inc., San Diego, California, USA (V. Brès, D. Ocampo, J.M. Linnen);; RTI International, Rockville, Maryland, USA (D.J. Brambilla);; Puerto Rico Department of Health, San Juan, Puerto Rico, USA (K.R. Ryff);; Banco de Sangre de Servicios Mutuos, San Juan, Puerto Rico, USA (E. Velez, G. Latoni);; Centers for Disease Control and Prevention, Fort Collins, Colorado, USA (L.R. Petersen);; Creative Testing Solutions, Tempe, Arizona, USA (P.C. Williamson)

**Keywords:** chikungunya virus, viruses, viremia, blood donors, epidemic, seroprevalence, nucleic acid amplification test, minipools, Puerto Rico

## Abstract

Chikungunya virus (CHIKV) caused large epidemics throughout the Caribbean in 2014. We conducted nucleic acid amplification testing (NAAT) for CHIKV RNA (n = 29,695) and serologic testing for IgG against CHIKV (n = 1,232) in archived blood donor samples collected during and after an epidemic in Puerto Rico in 2014. NAAT yields peaked in October with 2.1% of donations positive for CHIKV RNA. A total of 14% of NAAT-reactive donations posed a high risk for virus transmission by transfusion because of high virus RNA copy numbers (10^4^–10^9^ RNA copies/mL) and a lack of specific IgM and IgG responses. Testing of minipools of 16 donations would not have detected 62.5% of RNA-positive donations detectable by individual donor testing, including individual donations without IgM and IgG. Serosurveys before and after the epidemic demonstrated that nearly 25% of blood donors in Puerto Rico acquired CHIKV infections and seroconverted during the epidemic.

## Abstract 2

Deaths were rarely observed, but newborns and other vulnerable populations are at risk for severe complications.

Chikungunya virus (CHIKV), a mosquitoborne, positive-sense RNA virus of the family *Togaviridae*, causes an acute febrile illness and severe polyarthralgia that can persist for months or years in some patients (*1*–*3*). Serious outcomes and deaths are rarely observed. However, newborns and other vulnerable populations are at risk for severe complications (*4*).

In late 2013, cases of CHIKV infection were reported in the French Collectivity of Saint Martin, which is part of the French Antilles (*5*), constituting the first instance of autochthonous transmissions of CHIKV in the Americas in the past century (*6*). In an immunologically naive population, CHIKV spread rapidly throughout the Caribbean region and beyond to most countries in the Western Hemisphere (*7*), including 11 autochthonous cases reported in Florida, USA, in September 2014 (*8*).

CHIKV has yet to be demonstrated to be transmissible by blood transfusion (*9*). However, this finding might result from difficulties in discriminating transfusion transmission from locally acquired mosquitoborne infection. Transfusion transmission is probable, given previous instances of laboratory-acquired infections and infection of healthcare workers by blood exposures (*10*). Asymptomatically infected persons can have viral loads >10^5^ PFU/mL (*11*,*12*) and are a substantial risk for transfusion transmission.

Estimates of asymptomatic CHIKV infection vary widely. A recent study in Puerto Rico (*13*) confirmed previous estimates that 10%–25% of total infections are subclinical (*14*–*16*). However, other studies with the Asian genotype suggest that a greater proportion of cases might be asymptomatic or have only mild and transient symptoms (*17*,*18*). CHIKV infection can result in viral loads >10^8^ PFU/mL (*19*). Thus, relatively high viral loads likely present in some presymptomatic donors might be a threat for transfusion transmission. Recently, a case of transfusion transmission of the related alphavirus Ross River virus, has been reported (*20*), stemming from transfusion of the erythrocyte component from a blood donor who reported symptoms of Ross River virus infection 2 days after donating blood.

To mitigate the theoretical risk for transmission, some blood collection organizations in regions with large CHIKV epidemics have suspended local blood collection, implemented nucleic acid amplification testing (NAAT) of erythrocyte and plasma donations for CHIKV RNA, and introduced pathogen-reduction technology for platelet components (*21*,*22*). To directly assess the threat that CHIKV poses to the blood supply, and given the absence of licensed NAAT for donor screening, we conducted NAAT surveys of blood donors in Puerto Rico during the 2014 epidemic and complementary serosurveys before and after the epidemic.

## Materials and Methods

### Human Subjects Research Approval

We performed retrospective testing of anonymous blood donor samples and minipools. The study was approved by the University of California, San Francisco Committee for Human Research.

### Specimens

Creative Testing Solutions (Tempe, AZ, USA) retained, aliquoted, and archived at −70°C residual plasma from EDTA-anticoagulated blood collected in Puerto Rico and supplied for routine blood donor screening during the second half of 2014 and for a brief period during March 2015. Current molecular testing procedures at Creative Testing Solutions require that plasma samples be pooled into a minipool of 16 donor samples. Minipools prepared from blood donations in Puerto Rico were frozen during June 20–December 31, 2014. The sample set consisted of 1,667 minipools representing 26,672 individual donation samples from donors in Puerto Rico. Minipools were irreversibly stripped of their original labels and given a unique bar code that was linked only to month of collection.

In addition, 3,007 individual donor samples (IDS) were collected during the epidemic (September–November 2014), and ≈1,000 samples were saved per month. IDS were irreversibly stripped of all identifying information and given a unique bar code. Only basic demographic data (donor’s age, race, sex, county of residence, and week of collection) were retained in a secure database. Anonymous minipools and individual donor samples were retained, aliquoted, frozen, and stored at −70°C.

Finally, we retained 1,031 individual donation samples obtained during March 1–9, 2015, for a postepidemic serosurvey. Demographic data, including the donor’s age, sex, and zip code of residence, but not individual donor identifiers, were retained for these samples to enable analysis of serologic test results by using demographic strata.

### Viral RNA Testing

We performed viral RNA testing by using a prototype real-time CHIKV/dengue virus (DENV) target-capture, transcription-mediated amplification (TC-TMA) assay (*12*) (Hologic, Inc., San Diego, CA, USA). Plasma samples (0.5 mL) were tested by using the fully automated Panther System (Hologic, Inc.), which performs target capture, amplification, and real-time detection in the presence of an internal control. We achieved detection by using single-stranded, fluorescent-labeled nucleic acid probes that were present during amplification of the target. The time for the fluorescent signal to reach a specified threshold was proportional to the starting CHIKV and DENV RNA concentrations. Target capture oligonucleotides, TMA primers, and detection probes hybridize with highly conserved regions of CHIKV or DENV RNA genomes and were designed to detect all 3 major CHIKV lineages and all 4 DENV types. We set the cutoff value for reactive specimens at 1,000 relative fluorescent units.

Estimated viral loads for CHIKV were calculated relative to the emergence time of the emitted fluorescence of a calibration curve generated by testing logarithmic dilutions of a CHIKV in vitro–synthesized transcript. ID-NAAT–reactive specimens were diluted 1:16 in defribrinated, delipidated, pooled plasma (SeraCare, Gaithersburg, MD, USA) to mimic minipool testing and tested by TC-TMA assay to assess whether donation samples detected by ID-NAAT would have been detectable by minipool NAAT (MP-NAAT).

We determined limits of detection (LODs) by using an in vitro transcript corresponding to each analyte and calculation by using Enterprise Guide 5.1 Probit analysis and the Normal model (SAS Institute, Cary, NC, USA). For DENV-1–4, the 50% LOD was 1.7–2.1 copies/mL, and the 95% LOD was 7.1–13.0 copies/mL in the IDS format. For CHIKV, the 50% LOD was 4.6 copies/mL, and the 95% LOD was 19.7 copies/mL in the IDS format. In 16-member minipools for DENV-1–4, the 50% LOD was from 27.2–33.6 copies/mL, and the 95% LOD was 116.8–208.0 copies/mL. For CHIKV, the 50% LOD was 73.6 copies/mL, and the 95% LOD was 315.2 copies/mL in the MP format.

### Serologic Analysis

Plasma samples were tested for CHIKV IgM or IgG by using 2 ELISAs (Euroimmun US, LLC, Morris Plains, NJ, USA). These CHIKV ELISAs had specificities of 82% and 95% and sensitivities of 85% and 88% for IgM and IgG, respectively, when compared with those for 2 established in-house assays (*23*). Samples were diluted 1:100 and tested in duplicate according to the manufacturer’s instructions. Sample-to-calibrator ratios were calculated. In validating the assay, we found that preepidemic samples (n = 201) yielded no strongly positive samples when the manufacturer’s cutoff value >1.1 sample-to-calibrator ratio was used. However, 5 samples showed borderline reactivity (sample-to-calibrator ratios 1.13–1.37).

These 5 samples did not show positive results by reflex IgM testing, plaque-reduction neutralization testing (PRNT), or Western blot analysis when cell culture–propagated virus (strain 99659) was used as antigen. Testing of randomly chosen highly and moderately IgG-reactive samples from March 2015 by PRNT showed strong neutralization in all instances. Thus, the assay does not appear to yield strongly reactive false-positive results, but might yield a small frequency (5/201, 2.5%) of low-level reactive false-positive results. Therefore, a new cutoff value was established by using mean sample-to-calibrator ratios of preepidemic samples plus 5 SDs (1.42). Testing of multiple IgG-negative samples from both sample sets by IgM ELISA (20 samples), PRNT (20 samples), and Western blot analysis (10 samples) did not yield any suspected false-negative results, which suggested that false-negative results were also not common.

### Estimation of Detection Periods for MP-NAAT and IDS-NAAT

On the basis of the estimate for incidence of infection during the 2014 epidemic derived from serosurveys and MP-NAAT–positive results for the study period, we derived an estimate for duration of viremia detectable by the CHIKV TMA NAAT applied to minipools by using the approach of Busch et al. (*24*). We estimated the number of NAAT-positive donations in each minipool from minipool-testing results by using a program developed at the Centers for Disease Control and Prevention (Atlanta, GA, USA) (*25*). If T_i_ is the proportion of NAAT-positive donations in month i and P is seroprevalence of CHIKV at the end of the epidemic, then the TMA detection interval of CHIKV virus RNA (W) is estimated as 

Confidence limits for W were estimated by using a delta method estimate of the variance of W. Estimates for length of the individual donor sample-positive detection periods preceding and following the MP-NAAT–detectable period were derived from results of screening 3,007 individual donor samples by using ratios of samples detectable only by ID-NAAT that lacked IgG or contained IgG relative to the number of samples detectable at a dilution of 1:16. Confidence limits for these detection periods were derived by bootstrapping the assay results ratios (2/21) and (33/21) to obtain their variances, and then combining those with the variance associated with the estimate for the minipool detection period to obtain the variance of each of the 2 window estimates.

## Results

Of 1,668 minipools tested, 1 was positive for DENV RNA, and 161 (9.7%) were positive for CHIKV RNA (Table 1). This finding indicates a minimum MP-NAAT–detectable infection rate of 0.6% (161 positive donations of 26,688 total donations), assuming only 1 of the 16 donations in each positive minipool was viremic. However, because the reactive minipool proportion peaked at 19.5% in September 2014 (Table 1), some pools would probably contain >1 viremic donation.

**Table 1:** Nucleic acid amplification testing for chikungunya virus in minipools of blood donations during a chikungunya epidemic, Puerto Rico, USA, 2014

Month	No. reactive minipools/no. tested (%)	Infection rate* (upper limit), %
June	0/106 (0.0)	0.0 (0.00)
July	8/193 (4.1)	0.26 (0.50)
August	26/293 (8.9)	0.58 (0.83)
September	51/262 (19.5)	1.34 (1.75)
October	57/299 (19.1)	1.31 (1.69)
November	12/243 (4.9)	0.32 (0.54)
December	7/272 (2.6)	0.16 (0.32)
Total	161/1,668 (9.7)	0.65 (0.93)

*In individual donors on the basis of minipools of 16 samples.

**Figure 1:**
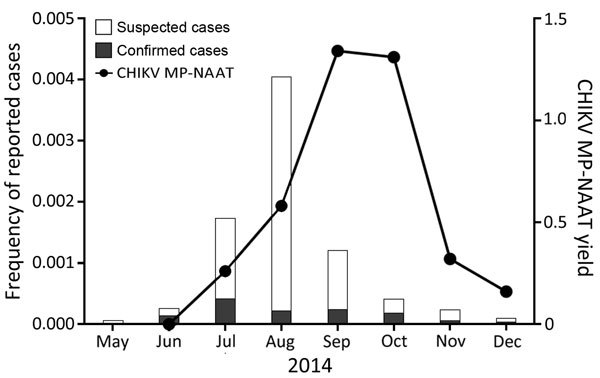
Estimated percentage of blood donations positive for chikungunya virus (CHIKV) RNA during a chikungunya epidemic, Puerto Rico, USA, 2014. CHIKV RNA-positive minipools of 16 donors were used to estimate the percentage of positive donations for the last 7 months of 2014. Estimates were made by using an algorithm for calculating infection rates from pooled data. Data from the Puerto Rico Department of Health for reported (suspected) and confirmed chikungunya case reports was used to transform data into estimated frequency of reported cases in a population in Puerto Rico of ≈3,548,400. MP-NAAT, minipool nucleic acid amplification testing.

Individual donations comprising reactive minipools were not archived for further testing. Thus, we could not directly determine numbers of reactive IDS per reactive minipool. Therefore, we used a published algorithm (*25*) to estimate the proportion of donations that would contain CHIKV RNA at levels detectable by MP-NAAT (Table 1). This modification yielded an estimate for MP-NAAT detectable viremia of 0.65% for the overall season and an upper limit of 0.93%. The highest estimated proportion of MP-NAAT–detectable CHIKV RNA-positive donations was during September and October (1.34% and 1.31% of donations reactive for CHIKV RNA by MP-NAAT, respectively) (Table 1). This estimation represented a slightly delayed peak when compared with suspected and confirmed clinical cases reported in Puerto Rico (Figure 1).

**Figure 2:**
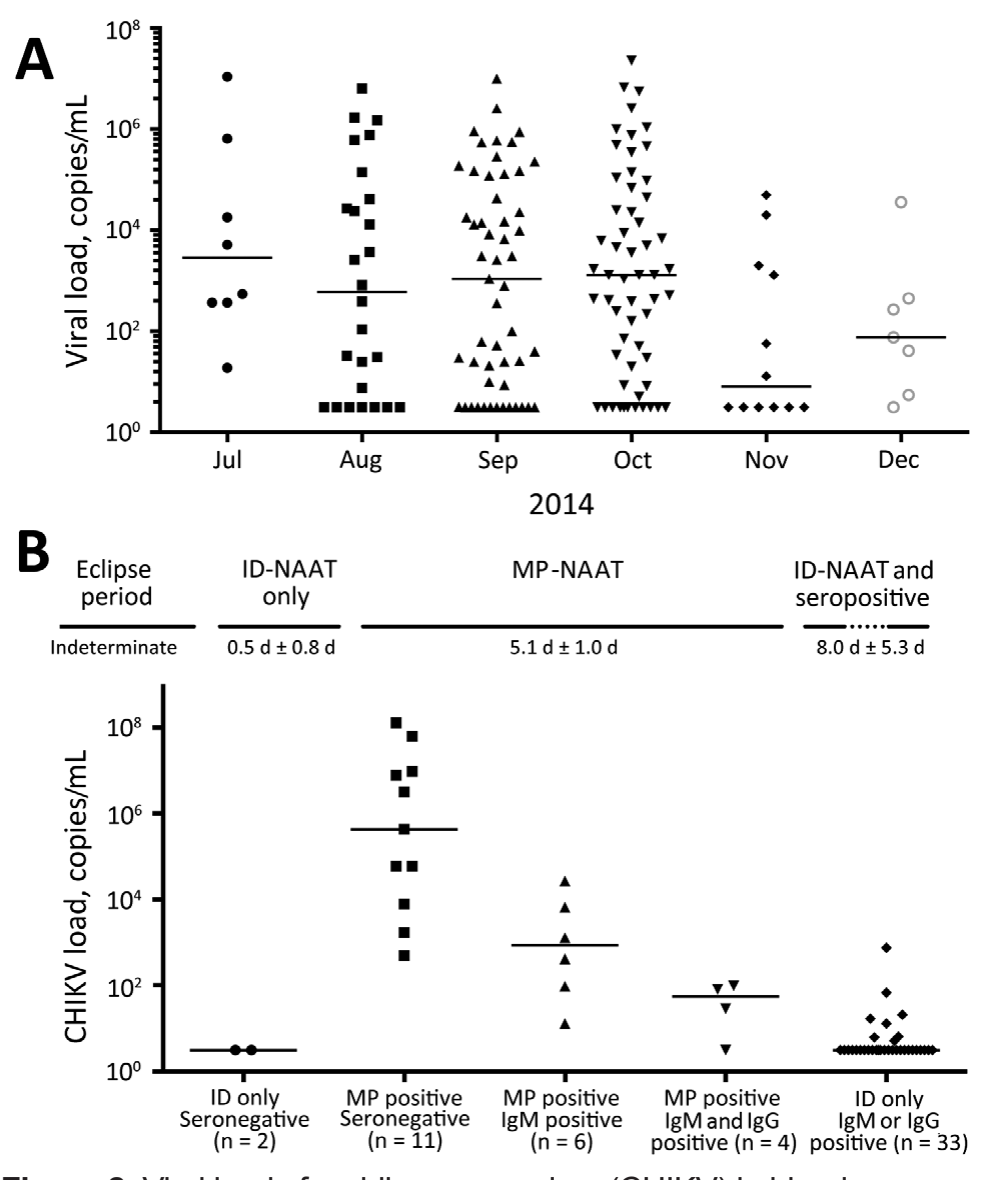
Viral loads for chikungunya virus (CHIKV) in blood donations during a chikungunya epidemic, Puerto Rico, USA, 2014. A) Positive minipool (MP) viral loads. Estimated viral loads (RNA copies/mL) were calculated for each reactive MP identified by using target capture transcription-mediated amplification (TC-TMA) during the epidemic. June 2014 (n = 106) is not plotted because of a lack of positive samples. Positive samples with unquantifiable viral loads are plotted as being at the limit of quantification (3.16 copies/mL) and were included in calculation of medians (horizontal bars). B) Individual donor (ID) viral loads for CHIKV. Estimated viral loads were calculated for each positive specimen identified by using TC-TMA during the 3 peak months of the epidemic. Positive samples with unquantifiable viral loads are plotted as being at the limit of quantification (3.16 copies/mL) and were included in calculation of medians (horizontal bars). Samples are arranged in order of projected time postinfection on the basis of predicted time course of acute infection (shown as estimated mean ±SD time intervals in days). ID only, samples positive by nucleic acid amplification testing (NAAT) but not positive for a 1:16 dilution mimicking minipooling. MP positive, samples positive by ID-NAAT and at a 1:16 dilution. Dynamics of acute infection with CHIKV (*26*) from the eclipse period (negative for virus RNA and IgM and IgG against CHIKV) to the end of infection (positive or negative for virus RNA and positive for IgM and IgG against CHIKV) is based on similar staging of dynamics of acute infection for other arboviruses (*27*) and approximate detection periods as described in the text.

Although not optimized to be quantitative, the TC-TMA assay provided approximate viral RNA copy numbers (Figure 2, panel A). Several minipools, particularly from early in the epidemic, had >10^7^ copies/mL, although they were tested as a minipool, and thus effectively diluted 1:16. Of 161 reactive minipools, 125 had quantifiable viral loads. Remaining minipools had viral loads less than an estimated value of 0.5 log copies/mL (according to the calibration curve). The median viral load of 161 reactive minipools was 550 copies/mL (range <3.16 copies/mL–2.3 × 10^7^ copies/mL). Donations from November and December had lower viral loads than donations from preceding months.

We also performed testing of archived IDS for CHIKV RNA for 3,007 donations collected in Puerto Rico during September–November 2014. We identified 56 confirmed positive donations, and ID-NAAT yields were 1.7%–2.1% for the 3 months tested (Table 2). When samples were diluted 1:16 to mimic minipools, proportions of RNA-positive samples detectable by MP-NAAT for September–November decreased to 0.4%–0.9%. Only 21 (37.5%) of 56 ID-NAAT–reactive specimens were reactive when tested for CHIKV RNA at a dilution of 1:16. Thus, 35 (62.5%) of 56 specimens would probably have been missed by routine MP-NAAT (Table 2). As expected, viral loads were low in donations reactive only by ID-NAAT. Only 8 of the ID-NAAT only–reactive samples had quantifiable viral loads (range 5.2–760 copies/mL) (Figure 2, panel B).

**Table 2:** Individual blood donations tested for chikungunya virus by nucleic acid amplification testing and serologic analysis during a chikungunya epidemic, Puerto Rico, USA, 2014*

Month	No. samples	No. ID-NAAT reactive samples	ID-NAAT yield, %	No. reactive at 1:16 dilution (MP-NAAT)	IgM reactive		IgG reactive	
					Total	IgM+/ID-only reactive	Total	IgG+/ID-only reactive
September	987	18	1.8	8	11	7†	8	7‡
October	1,010	21	2.1	9	15	10	14	10
November	1,010	17	1.7	4	16	12	14	12
Total	3,007	56	1.9	21	42	32†	36	32‡

*ID, individual donor; NAAT, nucleic acid amplification testing; MP, minipool. †Includes 1 IgM-positive/IgG-negative ID-only positive specimen. ‡Includes one IgM-negative/IgG-positive ID-only positive specimen.

We performed assays to detect IgM and IgG in the 56 ID-NAAT–reactive specimens to characterize the relationship between development of IgG and IgM, viral load, and the ability of minipool testing to detect viremic donations (Table 2). Thirteen (23.2%) of 56 samples were seronegative; 2 were detectable only by ID-NAAT. These 2 samples are presumed to represent donors detected in the earliest stages of acute infection. The remaining 11 seronegative viremic donations had detectable viral loads (range 5 ×10^2^–1.3 × 10^8^ copies/mL) (Figure 2, panel B), including 8 (14.3%) of 56 with viral loads >10^4^ copies/mL. These samples were probably from donors who were near the peak of viremia, but still collected before seroconversion occurred.

Most CHIKV RNA-reactive samples were IgM positive (75%) and IgG positive (64%); 1 sample was IgM negative and IgG positive. Development of IgG titers is an inverse correlate of CHIKV RNA detection (*28*); of the IgG-reactive samples, only 4 (11.1%) of 36 were detectable by the less sensitive MP-NAAT. Viral loads of samples sorted on the basis of NAAT results (ID only vs MP-NAAT detectable) and serologic data demonstrate a typical profile of acute viral infection (Figure 2, panel B). The 43 viremic IgM-positive or IgG-positive donations had significantly lower viral loads (median <3.16 copies/mL) than 13 viremic seronegative donations (60,000 copies/mL; p<0.0001 by 2-tailed Mann-Whitney test). Although similar proportions of ID-NAAT–positive samples were detected in November (1.7%) and September (1.8%), only 2 (11.8%) of 17 were seronegative in November compared with 6 (33.3%) of 18 in September, which suggested waning of the epidemic and a higher proportion of donations at the end of acute infection.

To estimate the incidence of CHIKV infection during the 2014 epidemic, we performed IgG serologic studies on blood donor specimens collected at the beginning of the epidemic (June 2014; preepidemic) and after the epidemic had subsided (March 2015; postepidemic). Collection was delayed until March to maximize detection of IgG seroconversion and to enable the maximum period for potential donors to recover from symptomatic infection, which would result in self-deferral, or deferral by the blood collection organization.

**Figure 3:**
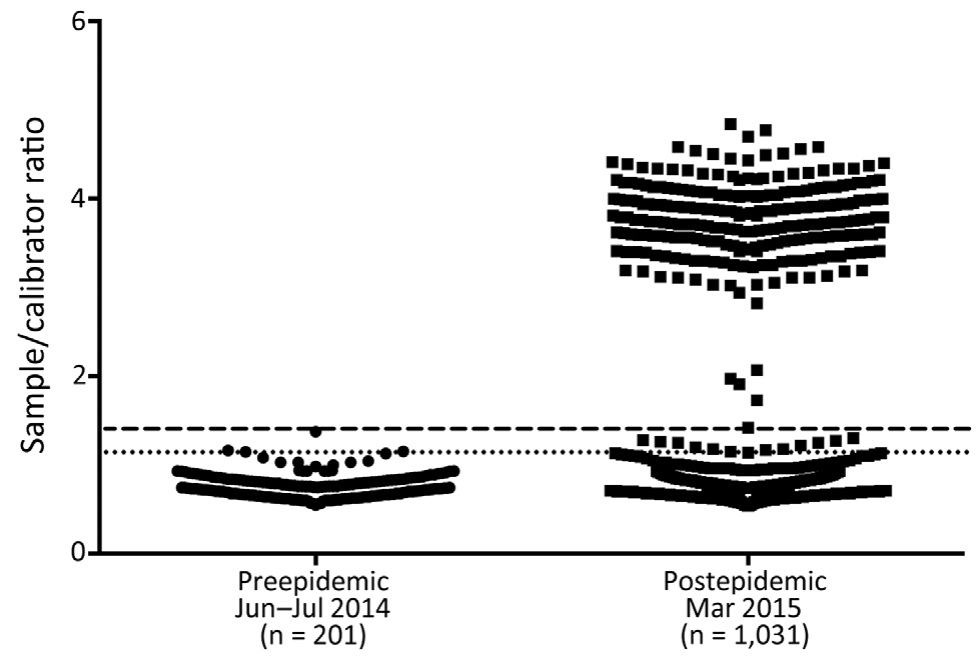
Serosurvey for chikungunya virus IgG in blood donations during a chikungunya epidemic, Puerto Rico, USA, 2014. Preepidemic samples collected in June and July 2014 were tested by using an IgG ELISA. A stringent cutoff value of mean + 5 SD (dashed line) was calculated from preepidemic samples. A less stringent cutoff value of mean + 3 SD (dotted line) was also calculated. These cutoff values were then applied to postepidemic samples collected in March 2015.

On the basis of IgG testing, we found that there were no unequivocally seroreactive samples in preepidemic samples (n = 201). In contrast, 241 (n = 1,031) postepidemic samples were strongly reactive (sample-to-calibrator ratio >2.5) (Figure 3). An additional indeterminate sample was positive by confirmatory testing with IgM ELISA, PRNT, and Western blot analysis. Thus, 242 (23.5%) of 1,031 samples were conservatively characterized as reactive (Figure 3).

Before we relabeled samples so that CHIKV testing was anonymous, basic demographic data were extracted for many of the specimens from March 2015 tested for seroreactivity (Table 3). No differences were observed in seropositivity rates between men and women. Persons 16–19 years of age had the highest rate of CHIKV recent infection; 40 (43.0%) of 93 of these persons were seropositive. In contrast, only 30 (18.3%) of 164 persons 40–49 years of age were seropositive.

**Table 3:** Demographic characteristics of blood donors tested for chikungunya virus during a chikungunya epidemic, Puerto Rico, USA, 2014

Characteristic	No. (%) nonreactive for IgG, n = 786*	No. (%) reactive for IgG, n = 242*	Total, n = 1,031*	Odds ratio (95% CI)
Sex				
** F**	235 (75.81)	75 (24.19)	310	1.00
** M**	348 (74.95)	117 (25.05)	567	1.05 (0.75–1.47)
Age, y				
** 16–19**	53 (56.99)	40 (43.01)	93	1.00
** 20–29**	139 (81.29)	32 (18.71)	171	0.31 (0.17–0.55)
** 30–39**	119 (79.33)	31 (20.67)	150	0.35 (0.19–0.62)
** 40–49**	134 (81.71)	30 (18.29)	164	0.30 (0.16–0.54)
** 50–59**	90 (70.54))	38 (29.46)	129	0.55 (0.31–0.97)
** 60–78**	49 (70.00)	21 (30.00)	69	0.57 (0.29–1.10)

*Some specimens did not have complete demographic data.

We combined results from MP-NAAT and ID-NAAT screening and the serosurvey to estimate lengths of time that CHIKV RNA is detectable in serial stages of viremia in asymptomatic donors by MP-NAAT and ID-NAAT used in this study (Figure 2, panel B). We estimated that the length of the MP-NAAT–detectable phase for acute CHIKV infection in asymptomatic persons who donated blood was 5.1 days (confidence limit 4.1–6.0 days). By applying the ratios of seronegative ID-NAAT–only donations (2/56), MP-NAAT–detectable donations (21/56), and ID-NAAT–only seropositive donations (33/56), we estimated that there is a transient stage of low viral load infection preceding viremia detectable by MP-NAAT (0.5 days; confidence limit 0–1.3 days), whereas there is a relatively long stage of persistent viremia after seroconversion (8 days; confidence limit 2.7–13.3 days).

## Discussion

Large epidemics of CHIKV infection occurred in the Caribbean Islands and in Central and South America over the past 2 years. Although >1.5 million confirmed and suspected cases have been reported (*29*), continued monitoring of CHIKV in these immunologically naive populations is needed for understanding population immunity and predicting dynamics of future epidemics. Using MP-NAAT, we estimated that 0.58% of individual blood donations were positive for CHIKV RNA during August 2014, a finding that is consistent with reported rates for Puerto Rico (*12*) and other Caribbean Islands (*22*).

As the 2014 epidemic in Puerto Rico continued, proportions of CHIKV viremia peaked in blood donors during September and October; >2% of donors were viremic, as indicated by individual donor NAAT results. During September and October, 1,440 chikungunya cases confirmed by real-time reverse transcription PCR were reported to the Puerto Rico Department of Health, which indicated sustained levels of CHIKV in the general population. However, reports of suspected chikungunya cases by month of illness onset received by the Puerto Rico Department of Health through passive surveillance peaked in August 2014 (Figure 1), which resulted in ≈14,000 suspected chikungunya cases in August, including 741 chikungunya cases confirmed by real-time reverse transcription PCR (Puerto Rico Department of Health, 2015, unpub. data). 

Several factors probably affect the relative frequency of viremia and seroincidence of CHIKV in blood donors compared with clinical cases documented in the general population, including the focal nature of the epidemic in Puerto Rico during 2014 in relation to blood donor center locations. It is also likely that many cases went unreported and that as the epidemic progressed many infected persons might not have sought medical care (*13*). Our finding that ≈25% of blood donors had serologic evidence of CHIKV infection after the 2014 epidemic supports these suggestions. Given a population of >3.5 million, and assuming that blood donors are representative of the total population of Puerto Rico with respect to risk for arbovirus transmission, a seroincidence of 23.5% would suggest that >800,000 persons were infected in Puerto Rico during the 2014 epidemic.

Blood safety protocols in place during the study included a Puerto Rico Department of Health requirement for questioning of donors concerning symptoms in the month preceding donation and passive reporting of postdonation febrile illness. Thus, in the absence of specific NAAT screening, asymptomatic donors are likely to result in most viremic donations (*30*).

It is not clear whether asymptomatic infection is correlated with lower viremia levels, and thus would decrease the likelihood of transfusion transmission. However, similar to previous findings (*12*), many presumably asymptomatic donors in our study had viral loads comparable with those for symptomatic patients (*11*,*19*), including some viral loads >10^8^ copies/mL. Most donations with low viral loads were IgM positive, which indicates recent acute infections. The proportion of these viremic specimens increased as the epidemic waned, and the percentage of ID-NAAT–only samples increased from 56% in September to 77% in November. Lower average viral copy numbers were also observed in November and December by testing of minipools. Furthermore, all RNA-positive donors in November were seropositive compared with only 78% of NAAT-reactive donors in September.

We estimate that the RNA-detectable window for MP-NAAT was 5.1 days. This value matches viremic periods observed for experimentally infected nonhuman primates (*31*) but is somewhat shorter than estimates for symptomatic patients of 1–2 days before disease onset and 8 days postonset (*9*,*11*,*32*,*33*). This finding is probably caused by a loss in the ability to detect viremia at the 1:16 dilution inherent in creating minipools, but might be a reflection that this study was limited to asymptomatic persons who donated blood. In addition, we calculated a relatively short ramp-up period before MP-NAAT–detectable viremia (0.5 days) and a longer low-level (MP-NAAT negative) viremia at the end of acute infection after seroconversion (8 days). Nevertheless, the 5-day MP-NAAT–detectable period for high-titer viremia is probably the most infectious period in terms of transfusion transmission and transmission to mosquitoes.

The overall threat CHIKV poses to the blood supply remains an open question that requires urgent attention, including in the continental United States, given the risk for travel-acquired and autochthonous transmission. In the absence of routine NAAT for CHIKV, and in regions where pathogen-reduction technology is not implemented, the largest threat is probably from donors with high viral loads who have not fully seroconverted because it can be assumed that donors with neutralizing IgG responses have a lower probability of transmitting an infectious dose to a recipient.

Although convalescent-phase serum is protective in animal studies (*34*), the ability of IgM and IgG in viremic donors to mitigate CHIKV transfusion transmission requires further study. Likewise, if viral RNA screening is introduced, studies will be needed to evaluate the relative usefulness of ID versus MP-NAAT. In screening of 3,007 individual donations, we identified 7 viremic donors with only IgM responses. However, only 1 of these donors had viremia detected only by ID-NAAT. We also identified 2 seronegative donors who showed reactivity by ID-NAAT, but not minipool testing. Whether blood components from these donations, together with specimens in the so-called eclipse phase between acquisition of infection and detectable ID-NAAT reactivity, are infectious remains unanswered.

In summary, our results indicated a sizable proportion of blood donors had detectable CHIKV RNA during the chikungunya epidemic in Puerto Rico in 2014. Several donations with high viremias were negative for IgM and IgG, which suggested that donors were in the peak phase of acute infection and highlights the risk for transfusion transmission. However, most viremic donations had low levels of viral RNA and were seropositive, which suggests recent subclinical infection and low risk for infectivity. However, these donors were healthy enough to donate blood. Finally, serosurveys before and after peak epidemic months showed that ≈25% of blood donors in Puerto Rico acquired CHIKV during the 2014 epidemic. On the basis of findings of this study, we are now conducting further investigations to determine the risk for transfusion transmission of CHIKV by virus RNA–positive transfusions and outcomes of infection in recipients.

## References

[1] Charrel RN, de Lamballerie X, Raoult D. Chikungunya outbreaks: the globalization of vectorborne diseases. N Engl J Med. 2007;356:769–71. 10.1056/NEJMp078013 17314335

[2] Pialoux G, Gauzere BA, Jaureguiberry S, Strobel M. Chikungunya, an epidemic arbovirosis. Lancet Infect Dis. 2007;7:319–27. 10.1016/S1473-3099(07)70107-X 17448935

[3] Powers AM, Logue CH. Changing patterns of chikungunya virus: re-emergence of a zoonotic arbovirus. J Gen Virol. 2007;88:2363–77. 10.1099/vir.0.82858-0 17698645

[4] Rampal SM, Meena H. Neurological complications in chikungunya fever. J Assoc Physicians India. 2007;55:765–9 . 18290551

[5] Cassadou S, Boucau S, Petit-Sinturel M, Huc P, Leparc-Goffart I, Ledrans M. Emergence of chikungunya fever on the French side of Saint Martin Island, October to December 2013. Euro Surveill. 2014;19:20752. 10.2807/1560-7917.ES2014.19.13.20752 24721536

[6] Halstead SB. Reappearance of chikungunya, formerly called dengue, in the Americas. Emerg Infect Dis. 2015;21:557–61. 10.3201/eid2104.141723 25816211 PMC4378492

[7] Rolph MS, Foo SS, Mahalingam S. Emergent chikungunya virus and arthritis in the Americas. Lancet Infect Dis. 2015;15:1007–8. 10.1016/S1473-3099(15)00231-5 26333330

[8] Kendrick K, Stanek D, Blackmore C; Centers for Disease Control and Prevention. Notes from the field: transmission of chikungunya virus in the continental United States—Florida, 2014. MMWR Morb Mortal Wkly Rep. 2014;63:1137 . 25474035 PMC4584604

[9] Petersen LR, Epstein JS. Chikungunya virus: new risk to transfusion safety in the Americas. Transfusion. 2014;54:1911–5. 10.1111/trf.12790 25130331 PMC6482831

[10] Cordel H, Quatresous I, Paquet C, Couturier E. Imported cases of chikungunya in metropolitan France, April 2005–February 2006. Euro Surveill. 2006;11:E060420.3 . 16809828 10.2807/esw.11.16.02944-en

[11] Appassakij H, Khuntikij P, Kemapunmanus M, Wutthanarungsan R, Silpapojakul K. Viremic profiles in asymptomatic and symptomatic chikungunya fever: a blood transfusion threat? Transfusion. 2013;53:2567–74. 10.1111/j.1537-2995.2012.03960.x 23176378

[12] Chiu CY, Bres V, Yu G, Krysztof D, Naccache SN, Lee D, Genomic assays for identification of chikungunya virus in blood donors, Puerto Rico, 2014. Emerg Infect Dis. 2015;21:1409–13. 10.3201/eid2108.150458 26196378 PMC4517739

[13] Sharp TM, Roth NM, Torres J, Ryff KR, Perez Rodriguez NM, Mercado C, Chikungunya cases identified through passive surveillance and household investigations—Puerto Rico, May 5–August 12, 2014. MMWR Morb Mortal Wkly Rep. 2014;63:1121–8 . 25474032 PMC4584601

[14] Sissoko D, Moendandze A, Malvy D, Giry C, Ezzedine K, Solet JL, Seroprevalence and risk factors of chikungunya virus infection in Mayotte, Indian Ocean, 2005–2006: a population-based survey. PLoS ONE. 2008;3:e3066. 10.1371/journal.pone.0003066 18725980 PMC2518850

[15] Moro ML, Gagliotti C, Silvi G, Angelini R, Sambri V, Rezza G, Chikungunya virus in North-Eastern Italy: a seroprevalence survey. Am J Trop Med Hyg. 2010;82:508–11. 10.4269/ajtmh.2010.09-0322 20207883 PMC2829919

[16] Queyriaux B, Simon F, Grandadam M, Michel R, Tolou H, Boutin JP. Clinical burden of chikungunya virus infection. Lancet Infect Dis. 2008;8:2–3. 10.1016/S1473-3099(07)70294-3 18156079

[17] Nakkhara P, Chongsuvivatwong V, Thammapalo S. Risk factors for symptomatic and asymptomatic chikungunya infection. Trans R Soc Trop Med Hyg. 2013;107:789–96. 10.1093/trstmh/trt083 24052594

[18] Yoon IK, Alera MT, Lago CB, Tac-An IA, Villa D, Fernandez S, High rate of subclinical chikungunya virus infection and association of neutralizing antibody with protection in a prospective cohort in the Philippines. PLoS Negl Trop Dis. 2015;9:e0003764. 10.1371/journal.pntd.0003764 25951202 PMC4423927

[19] Chow A, Her Z, Ong EK, Chen JM, Dimatatac F, Kwek DJ, Persistent arthralgia induced by chikungunya virus infection is associated with interleukin-6 and granulocyte macrophage colony-stimulating factor. J Infect Dis. 2011;203:149–57. 10.1093/infdis/jiq042 21288813 PMC3071069

[20] Hoad VC, Speers DJ, Keller AJ, Dowse GK, Seed CR, Lindsay MD, First reported case of transfusion-transmitted Ross River virus infection. Med J Aust. 2015;202:267–70. 10.5694/mja14.01522 25758699

[21] Rasonglès P, Angelini-Tibert MF, Simon P, Currie C, Isola H, Kientz D, Transfusion of platelet components prepared with photochemical pathogen inactivation treatment during a chikungunya virus epidemic in Ile de La Reunion. Transfusion. 2009;49:1083–91. 10.1111/j.1537-2995.2009.02111.x 19309473

[22] Gallian P, de Lamballerie X, Salez N, Piorkowski G, Richard P, Paturel L, Prospective detection of chikungunya virus in blood donors, Caribbean 2014. Blood. 2014;123:3679–81. 10.1182/blood-2014-03-564880 24904107

[23] Prat CM, Flusin O, Panella A, Tenebray B, Lanciotti R, Leparc-Goffart I. Evaluation of commercially available serologic diagnostic tests for chikungunya virus. Emerg Infect Dis. 2014;20:2129–32. 10.3201/eid2012.141269 25418184 PMC4257799

[24] Busch MP, Wright DJ, Custer B, Tobler LH, Stramer SL, Kleinman SH, West Nile virus infections projected from blood donor screening data, United States, 2003. Emerg Infect Dis. 2006;12:395–402. 10.3201/eid1205.051287 16704775 PMC3291460

[25] Biggerstaff BJ. Confidence intervals for the difference of two proportions estimated from pooled samples. J Agric Biol Environ Stat. 2008;13:478–96. 10.1198/108571108X379055

[26] Schwartz O, Albert ML. Biology and pathogenesis of chikungunya virus. Nat Rev Microbiol. 2010;8:491–500. 10.1038/nrmicro2368 20551973

[27] Busch MP, Kleinman SH, Tobler LH, Kamel HT, Norris PJ, Walsh I, Virus and antibody dynamics in acute West Nile virus infection. J Infect Dis. 2008;198:984–93. 10.1086/591467 18729783

[28] Prince HE, Seaton BL, Matud JL, Batterman HJ. Chikungunya virus RNA and antibody testing at a national reference laboratory since the emergence of chikungunya virus in the Americas. Clin Vaccine Immunol. 2015;22:291–7. 10.1128/CVI.00720-14 25540275 PMC4340891

[29] Pan American Health Organization. Number of reported cases of chikungunya fever in the Americas—epidemiological week 45, 2015 [cited 2016 Apr 4]. http://www.paho.org/hq/index.php?option=com_docman&task=doc_download&Itemid=270&gid=32212&lang=en.

[30] Appassakij H, Promwong C, Rujirojindakul P, Wutthanarungsan R, Silpapojakul K. The risk of blood transfusion-associated chikungunya fever during the 2009 epidemic in Songkhla Province, Thailand. Transfusion. 2014;54:1945–52. 10.1111/trf.12575 24527811

[31] Messaoudi I, Vomaske J, Totonchy T, Kreklywich CN, Haberthur K, Springgay L, Chikungunya virus infection results in higher and persistent viral replication in aged rhesus macaques due to defects in anti-viral immunity. PLoS Negl Trop Dis. 2013;7:e2343. 10.1371/journal.pntd.0002343 23936572 PMC3723534

[32] Chusri S, Siripaitoon P, Silpapojakul K, Hortiwakul T, Charernmak B, Chinnawirotpisan P, Kinetics of chikungunya infections during an outbreak in Southern Thailand, 2008–2009. Am J Trop Med Hyg. 2014;90:410–7. 10.4269/ajtmh.12-0681 24493674 PMC3945684

[33] Lanciotti RS, Kosoy OL, Laven JJ, Panella AJ, Velez JO, Lambert AJ, Chikungunya virus in US travelers returning from India, 2006. Emerg Infect Dis. 2007;13:764–7. 10.3201/eid1305.070015 17553261 PMC2738459

[34] Couderc T, Khandoudi N, Grandadam M, Visse C, Gangneux N, Bagot S, Prophylaxis and therapy for chikungunya virus infection. J Infect Dis. 2009;200:516–23. 10.1086/600381 19572805 PMC7109959

